# Single-molecule study of oxidative enzymatic deconstruction of cellulose

**DOI:** 10.1038/s41467-017-01028-y

**Published:** 2017-10-12

**Authors:** Manuel Eibinger, Jürgen Sattelkow, Thomas Ganner, Harald Plank, Bernd Nidetzky

**Affiliations:** 1 Institute of Biotechnology and Biochemical Engineering, Graz University of Technology, Petersgasse 10-12/1, 8010 Graz, Austria; 2 Institute of Electron Microscopy and Nanoanalysis, Graz University of Technology, Steyrergasse 17, 8010 Graz, Austria; 3 Graz Centre for Electron Microscopy, Steyrergasse 17, A-8010 Graz, Austria; 4 Austrian Centre of Industrial Biotechnology, Petersgasse 14, 8010 Graz, Austria

## Abstract

LPMO (lytic polysaccharide monooxygenase) represents a unique paradigm of cellulosic biomass degradation by an oxidative mechanism. Understanding the role of LPMO in deconstructing crystalline cellulose is fundamental to the enzyme’s biological function and will help to specify the use of LPMO in biorefinery applications. Here we show with real-time atomic force microscopy that C1 and C4 oxidizing types of LPMO from *Neurospora crassa* (*Nc*LPMO9F, *Nc*LPMO9C) bind to nanocrystalline cellulose with high preference for the very same substrate surfaces that are also used by a processive cellulase (*Trichoderma reesei* CBH I) to move along during hydrolytic cellulose degradation. The bound LPMOs, however, are immobile during their adsorbed residence time ( ~ 1.0 min for *Nc*LPMO9F) on cellulose. Treatment with LPMO resulted in fibrillation of crystalline cellulose and strongly ( ≥ 2-fold) enhanced the cellulase adsorption. It also increased enzyme turnover on the cellulose surface, thus boosting the hydrolytic conversion.

## Abstract (web summary)

Understanding the role of enzymes in biomass depolymerization is essential for the development of more efficient biorefineries. Here, the authors show by atomic force microscopy the real-time mechanism of cellulose deconstruction by lytic polysaccharide monooxygenases.

## Introduction

Sustainable production of fuels, chemicals and materials from plant biomass represents a major effort of global importance^1^. The potential advantages, whether for diversifying the energy portfolios, decreasing emissions or supporting rural developments, are important for society in the long run^2^. The capability of biotechnology-based biorefineries to play a significant role depends on finding sustainable and cost effective ways of deconstructing the complex lignocellulosic composites in abundant biomass feedstocks like agricultural residues and forestry wastes^3, 4^. Most of the current biorefinery designs involve advanced biofuels, predominantly ethanol, produced from sugars released from the feedstock by enzymatic saccharification^5^. The high resistance of biomass polysaccharides, most notably that of cellulose, constitutes a main hurdle any viable process design must overcome^3, 4^. Besides effective pretreatment, enzyme efficiency in breaking down the cellulose is key. Enzyme systems for cellulose degradation are all mixtures of a core set of individual activities, typically hydrolases that use chain-end cleavage (cellobiohydrolases) or internal chain cleavage (endoglucanases) for cellulose depolymerization^6^. Because these activities work in synergy, the mixture’s overall efficiency is determined by its composition. Huge research efforts were already targeted at engineering of cellulase systems^6, 7^. A new mechanism of cellulose degradation via O_2_-dependent oxidative chain cleavage was discovered recently^8–11^. Thus was fueled the expectation of a truly disruptive improvement of enzymatic deconstruction efficiency resulting from a suitable combination of hydrolytically and oxidatively chain-cleaving activities. The relevant oxidative enzyme, lytic polysaccharide monooxygenase (LPMO), is included in the currently most advanced enzyme cocktails for cellulose saccharification^12^. However, deepened understanding of the effect of LPMO on deconstructing cellulosic material is urgently required to define the oxidative enzyme’s most appropriate use in biomass processing. Despite significant progress in characterizing LPMO structurally and mechanistically^9–13^, evidence on the behavior of LPMO on the actual site of its catalytic action–the solid cellulose surface–is limited. To advance this evidence is not only fundamental to the enzyme’s biological function in cellulose degradation but it will also help specifying the exploitation of LPMO in emergent biorefinery applications.

LPMO is a metalloenzyme with a mononuclear Cu^2+^ center in the active site^9, 11–14^. Catalysis depends on external supply of electrons for Cu^2+^ reduction, sources of which can be small-molecule redox mediators or partner redox-proteins^15–17^. Polysaccharide chain cleavage occurs with insertion of a single oxygen atom at C1 or C4 of an intrachain cellobiosyl moiety^10, 13^, depending on the type of LPMO used. LPMO structures suggest a likely binding mode of the enzyme to cellulose surfaces via the protein face that exposes the catalytic metal outward^9^. Oxidative chain cleavages in crystalline areas of the substrate are expected to cause local disruptions of the ordered cellulose structure^18, 19^. This decrystallization of the substrate might facilitate the hydrolytic chain depolymerization by cellulases. We previously used atomic force microscopy (AFM) to visualize the effect of LPMO on cellulose surface degradation^20^. We demonstrated that in amorphous-crystalline cellulose films, LPMO attacked preferably the crystalline substrate areas and effectively degraded small fibrils exposed on the surface. Treatment of the substrate with LPMO enabled cellulases to break down crystalline cellulose nanostructures, which is otherwise highly resistant to degradation.

Here we analyze the dynamic interaction of LPMO with crystalline cellulose in detail, applying real-time AFM with lateral and temporal resolutions suitable for tracking single enzyme molecules on the cellulose surface. Besides visualizing the LPMO, we study the effects on substrate degradation and show surface fibrillation to occur as a result of the LPMO action. We furthermore demonstrate the increased overall adsorption to cellulose and enhanced surface mobility of a well-characterized cellulase, the cellobiohydrolase I from *Trichoderma reesei*, in the presence of LPMO. We study two LPMOs from the fungus *Neurospora crassa*, one (*Nc*LPMO9F) representing the C1 and the other (*Nc*LPMO9C) the C4 oxidizing type of reactivity^21^. Both LPMOs are classified in the carbohydrate-active enzymes (CAZy) database into auxiliary activities (AA) family AA9^22^. Besides different chain cleavage mode, the two LPMOs also differ in that *Nc*LPMO9C contains a carbohydrate-binding module (CBM)^23^, whereas *Nc*LPMO9F is lacking one. The presence of a family 1 CBM, which is also present in many cellulases (e.g., CBH I)^6^, is of interest for it might affect how the enzyme binds to and processes further on the cellulose surface^24^.

## Results

**Fig. 1:**
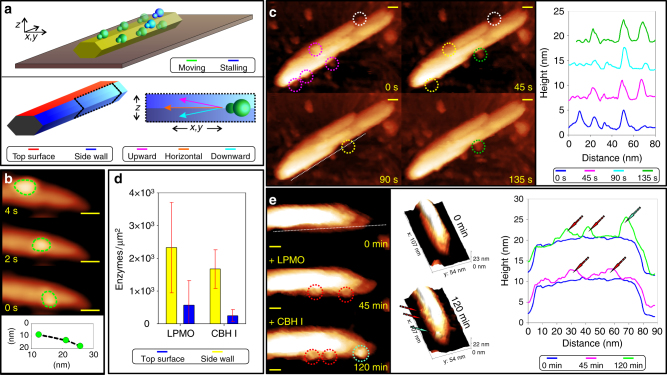
Single-molecule observations on cellulose nanocrystals. **a** Schematic of an idealized cellulose nanocrystal adsorbed to graphite and terminology for enzyme localization and movement on the cellulose. **b** Real-time AFM observation of CBH I molecules (outlined in color) on a cellulose nanocrystal. Images were recorded at 0.5 s^−1^ and the enzyme center was tracked to obtain the velocity data. The image sequence was taken from Supplementary Movie 2. **c** C1 oxidizing LPMO molecules on a cellulose nanocrystal visualized in real time (1.3 AFM frames min^−1^). Colored frames indicate enzymes present initially (magenta), adsorbing (green), or desorbing (vacated position; yellow) in the relevant timespan. The height profile along the dashed line shows multiple stationary LPMO molecules bound on the side wall of the cellulose nanocrystal. One LPMO molecule, circled white, was not included in the analysis, for it failed the rigorous height criterion. **d** Individual enzyme adsorption to the side walls (yellow bars) and to the top (blue bars) surface of cellulose nanocrystals. Mean values of enzymes-adsorbed/surface area of cellulose nanocrystal are shown together with the corresponding standard error. **e** Visualization and cross-sectional analysis of LPMO (circled red, red arrows) and CBH I (circled turquois, turquoise arrow) along the dashed line showed successive adsorption to the same cellulose nanocrystal. As enzymes differ in height (Supplementary Fig. 1), LPMOs ( ~  2 nm) are clearly distinguished from CBH I molecules ( ~ 4 nm). All scale bars are 10 nm

We initially analyzed the *T. reesei* CBH I for two reasons. First, Igarashi and colleagues^25^ previously showed the same CBH I to slide undirectionally along crystalline cellulose surfaces. The enzyme’s movement likely arose from its well-characterized processive mode of cellulose-chain degradation, involving successive cleavages of terminal cellobiose units from the same chain without intermediate chain release from enzyme^6^. Being able to track the movement of CBH I in our studies served to validate the general experimental setup as well as the temporal resolution of the AFM method used (Fig. 1). Second, in natural cellulase systems, of which the one from *T. reesei* is prototypical, the CBH I is mainly responsible for the degradation of crystalline cellulose^6^. Action of LPMO on the same cellulose material is therefore expected to influence the CBH I, strongest among the different enzymes present in the cellulase mixture. Evidence on the individual action of the CBH I was therefore required as a reference.

### Single-molecule analysis of LPMO and CBH I

To be able to identify LPMO and CBH I in single-molecule analysis by AFM, we performed an extensive height profile analysis of the enzymes adsorbed on the pyrolytic graphite used as grid in the experiments. With an average height of 1.5 ( ± 0.7)  nm, the LPMO was about twice smaller than CBH I (2.8 ± 1.1 nm), as shown in Supplementary Fig. 1. Due to differences in height, it was also possible to distinguish LPMO and CBH I one from another in an experiment using both enzymes at a time.

Figure 1a shows a schematic of an idealized cellulose nanocrystal attached to the graphite surface. It furthermore introduces the terminology for describing enzyme localization and movement on the cellulose nanocrystals. Analyzing 50 enzymes on five cellulose nanocrystals, we determined that roughly 10–30% of the adsorbed CBH I molecules were moving on the surface of cellulose and that they did so mostly on the side walls of the nanocrystals. An exemplary sequence of AFM images revealing CBH I molecules partly in movement is shown in Supplementary Movie 1. Side wall-adsorbed enzymes were initially revealed by visual inspection of the AFM images. Careful cross-sectional analysis confirmed their direct contact with the cellulose surface, with no gap left between enzyme and cellulose. An exemplary sliding motion of a CBH I molecule on the cellulose surface is shown in Fig. 1b. The figure was prepared from time-resolved AFM data in Supplementary Movie 2. The enzyme moved along the top surface, as clearly seen in a time-resolved trajectory analysis of the enzyme center, until the enzyme was desorbed. In general, the travel distance and the exact path of enzyme motion–upward, downward, or horizontal–varied depending on the crystal analyzed. The average velocity of continuous CBH I movement was measured as 3.1 ± 0.9 nm/s, consistent with the literature^26, 27^, however, unresolved into discrete stop and go phases, observed using higher time resolution^25, 28^.

Although LPMO is only about half the size of CBH I ( ~ 24 kDa), we were able to visualize the enzymes clearly on the crystalline cellulose, as shown in Fig. 1c. In stark contrast to CBH I, LPMO stayed at the place of its adsorption on the cellulose over several minutes. Of ≥ 400 enzyme molecules (C1 oxidzing LPMO) analyzed on 20 nanocrystals, none moved on the surface until it became eventually desorbed again. The C4′-oxidizing LPMO additionally equipped with CBM was also completely immobile on the cellulose surface (Supplementary Fig. 2). Clearly, therefore, LPMO is not a processively moving enzyme like CBH I is.

### Localization of LPMO and CBH I on the cellulose surface

CBH I and LPMO both showed pronounced ( ≥ 4-fold) preference for binding to the side walls of the cellulose nanocrystals, as compared to binding to the nanocrystals’ top surface. Figure 1d illustrates the effect, and counting single enzymes (75 molecules, 12 individual nanocrystals) provided quantitative results. Supplementary Fig. 1 was used to formulate a quantitative height criterion for the rigorous identification of LPMO molecules adsorbed to the side walls of cellulose, distinguishing these enzymes from other LPMOs bound to the cellulose and the surface of the graphite at the same time. A minimum of 2.5 nm was set for the height difference between the highest point of the LPMO molecule analyzed and the graphite support lying underneath. The height criterion was based on the enzyme’s average height of 1.5 nm. The corresponding height criterion used in the analysis of CBH I was 4.0 nm. By applying these criterions, we ensured that the enzymes analyzed for adsorption to the cellulose side walls, in the main ( ≥ 85%), did not involve additional contact with, and were therefore unaffected by, the graphite surface.

Comparing positions of the adsorbed enzymes based on their average footprint area on the cellulose surfaces, we find that LMPO bound to surface regions of the cellulose crystals (Fig. 1a) also used by CBH I for binding. Co-localization of LPMO and CBH I on the top surface of a cellulose crystal was furthermore shown in Fig. 1e. Overlapping specificity for adsorption to crystalline cellulose surfaces is therefore suggested for the two enzymes. The cellulose nanocrystals used represent cellulose polymorph I_β_, the main crystalline form of cellulose in the plant cell wall. Evidence from experimental and computational studies, reviewed by Payne et al.^6^ suggests that CBH I binds preferentially to the hydrophobic faces of cellulose I. The cellulose nanocrystals used have an aspect ratio of  ∼ 2.5 and an average width of 17.6 nm. Ideal nanocrystal morphology, which is consistent with these characteristics is shown in Fig. 1a. It involves a hexagon-like cross-section, and so it exhibits relatively broad hydrophobic faces. There is precedence for this particular type of morphology in cellulose nanocrystals produced from the acid hydrolysis of tunicates, as reviewed by Moon et al^29^. Orientation of such, ideally shaped, cellulose nanocrystals on the AFM grid with their broader surface in contact with the graphite surface would expose their presumably relevant hydrophobic faces on the side walls (Fig. 1a). Although in agreement with the evidence of enzymes adsorbing predominantly to the side walls of cellulose, these considerations should be taken cautiously, for cellulose morphology at this level of detail could not be revealed by measurement using AFM.

### Adsorption kinetics of LPMO

**Fig. 2:**
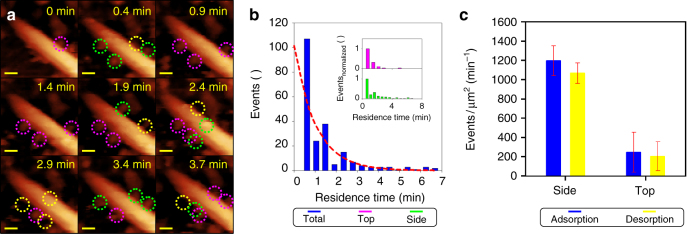
Adsorption/desorption of LPMO. **a** Real-time observation of C1 oxidizing LPMO molecules adsorbing to and desorbing from cellulose nanocrystals. Images are taken from Supplementary Movies 3 and 4. Colored frames show enzymes present initially (magenta), adsorbing (green), or desorbing (vacated position; yellow) in the relevant timespan. **b** The residence time of LPMO on the surface of nanocrystals. The histogram was fitted by an error weighted exponential decay function (shown in red), yielding a dissociation rate constant of 0.97 min^−1^. Qualitative analysis showed similar distributions for LPMOs independent of their adsorption site. **c** Adsorption and desorption events on the top and side surfaces of cellulose nanocrystals revealed by analyzing ~ 220 individual enzymes bound to 8 different nanocrystals. Mean values of events/surface area of cellulose nanocrystal are shown together with the corresponding standard error. Scale bars are 10 nm

We then monitored adsorption–desorption of single LPMO molecules in a time-resolved manner, as illustrated in Fig. 2a. The C1 oxidizing LPMO (*Nc*LPMO9F) was used. An average residence time of around 1 min was determined for LPMO bound to the cellulose surface, as shown in Fig. 2b. Comparison of top- and side-wall-associated LPMO molecules based on normalized residence time distributions revealed a comparable average residence time for both LPMO populations, as shown in Fig. 2b.

The on- and off-rate of LPMO binding to the crystalline cellulose was determined from the AFM data (~430 events, 8 microfibrils), as indicated in Fig. 2c. Both the on- and the off-rate were found to be about 4.5-fold higher on the side faces of the cellulose nanocrystals than on the crystals’ top face (Fig. 2c). The locally observed differences in protein adsorption dynamics are consistent with a preference of LPMO for binding to, hence attacking, the side surfaces of the nanocrystals. On the basis of the reported turnover numbers of LPMO (*k*_cat_ = 0.3–6 min^−1^)^21, 30, 31^, the average time of adsorption is sufficient to allow the bound enzyme to undergo between 0.3 and 6 catalytic events.

### LPMO-catalyzed oxidative deconstruction of cellulose nanocrystals

**Fig. 3:**
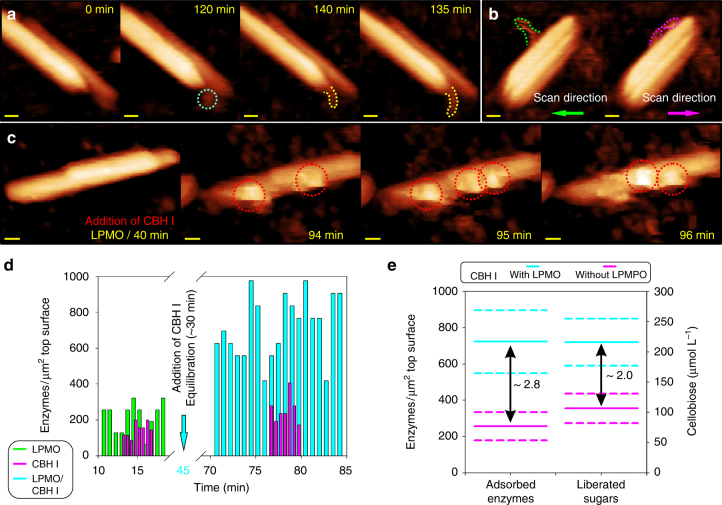
Real-time observations of LPMO activity on cellulose nanocrystals and its effect on CBH I. **a** Partial detachment of cellulose fibrils from nanocrystals as a result of C1 oxidizing LPMO activity. LPMO molecule adsorbs (blue circle) to an intact crystal and after desorption a fibril loosened from the crystalline material (yellow frame) becomes visible. **b** By comparing forward (trace; green frames) and backward direction (retrace; blue frames) of AFM line scans, the loosened parts of the fibrils are shown to be moveable by the AFM tip. The fibril part associated with the cellulose nanocrystal, by contrast, was not moveable. Further examples of cellulose nanocrystal fibrillation by LMPO are shown in Supplementary Fig. 3. **c** LPMO enhances the adsorption of CBH I to cellulose nanocrystals, as shown in AFM images (compare (**c**) and Fig. 1b) and quantitative single-molecule analysis (**d**). **d** The ratio of enzyme molecules/top surface area analyzed for 16 cellulose nanocrystals is shown for the conditions that LPMO acted alone and CBH I was added afterwards. Experiment using CBH I alone is shown as reference. Real-time observations are also shown in Supplementary Movies 5 and 6. Besides showing enhanced enzyme adsorption, the movies reveal an increased portion of the adsorbed enzymes to be mobile on the cellulose surface when LPMO and CBH I were present together. Supplementary Movie 2, which shows CBH I acting alone, is used as reference. **e** Synergy between CBH I and LPMO during degradation of cellulose nanocrystals is shown. LPMO addition stimulated the CBH I adsorption and resulted in increased release of soluble sugars. Solid lines represent mean values and standard error is indicated by dashed lines. All scale bars are 10 nm

Oxidative chain cleavages are expected to destabilize the spatial order of cellulose chains in crystalline material. Computational studies support the intuition^18^, but experimental evidence on the presumed deconstruction effect of LPMO action is lacking. Very recently^19^, solid-state NMR studies combined with AFM microscopy showed micron-sized cellulose fibers from bleached softwood to become globally disrupted upon incubation with LPMO. Here we were able to visualize local fibrillation events, which are occurring at the side walls of the cellulose nanocrystals, in consequence of treatment with LPMO. The partial detachment of cellulose chain bundles (fibrils) in enzymatically attacked nanocrystals is demonstrated in Fig. 3a. Additional data are shown in Supplementary Fig. 3a–c. Using careful comparison of the forward and backward direction of AFM line scans, we showed that the loosened parts of the fibrils were moveable in both directions by the AFM tip. Their anchoring points in the cellulose crystal, however, were completely unchanged in trace-retrace sequences of analysis. Therefore, this rigorously eliminated the possibility that the observed fibrils were not cellulose. Fibrillation occurred in about 10–20% of the 150 nanocrystals analyzed. Fibrillation was detectable only after extended incubation times in the presence of LPMO, suggesting it to represent a late stage in the overall substrate degradation by the enzyme.

### Synergy between LPMO and CBH I

A number of studies show that LPMO synergizes with CBH I in terms of enhanced saccharification of cellulose^5, 12, 20, 32–34^. On the basis of single-molecule visualization results, we succeeded in establishing a mechanistic basis of the synergistic effect. CBH I binding to the cellulose nanocrystals was strongly enhanced ( ≥ 2-fold) in the presence of LPMO, as shown in Fig. 3c–e. The increase in CBH I adsorption involved a somewhat enhanced binding at the side surfaces of the cellulose crystal, as shown in Supplementary Fig. 4. The largest part of it, however, was due to binding of CBH I at the top surface of the cellulose crystals. LPMO action appears to create adsorption sites for CBH I in crystalline cellulose surface, which is otherwise not accessible to the cellulase.

LPMO action also seems to invigorate the action of CBH I on the cellulose surface by enhancing the fraction of cellulase enzymes showing mobility. Observing individual enzyme molecules in time-resolved AFM sequences, we were able to show increased adsorption-desorption dynamics on the cellulose top surface when LPMO and CBH I both were present, as seen in Supplementary Movie 5. From a comparison of Supplementary Movies 2 and 6, it is recognized furthermore that not only the overall abundance but also the enzyme dynamics appeared to have been enhanced, when CBH I and LPMO were acting together as compared to CBH I acting alone. Enzymes recognized clearly as isolated particles, either mobile or stationary, were observed in both the presence and the absence of LPMO. However, in the presence of LPMO the dynamic formation of clusters comprising multiple individual enzymes in close physical proximity to each other was seen. Interestingly, these enzyme clusters appeared to be both stationary and mobile (Supplementary Movie 6), thus resembling to some extent the previously reported traffic jams^25^ of CBH I molecules on crystalline cellulose surfaces.

In general, enzyme processive mobility on the cellulose surface appeared to also have been increased in the presence of LPMO. The effect was difficult to quantify precisely due to the large number of individual enzyme molecules on the surface. However, the portion of adsorbed CBH I molecules detected in motion was clearly enhanced, reflected also by an apparent increase in the maximum speed of unidirectionally moving CBH I molecules, as shown in Supplementary Fig. 5. LPMO stimulated the CBH I adsorption independent of its addition before, after, or at the same time CBH I was added. Eventually this resulted in a 2-fold enhancement of the soluble sugar release by CBH I, as shown in Fig. 3e.

## Discussion

Single-molecule study of oxidative enzymatic deconstruction of nanocrystalline cellulose by C1 and C4 oxidizing types of LPMO (*Nc*LPMO9F, *Nc*LPMO9C) was performed. The dynamic features of LPMO adorption–desorption and enzyme behavior on the cellulose surface were revealed. Both LPMOs overlapped with CBH I in specificity for adsorption to the (likely hydrophobic) surfaces of the cellulosic substrate used. Contrary to CBH I that showed the canonical processive movement on the cellulose surface, the LPMOs appeared immobile during the time of their adsorption on cellulose. Besides releasing oxidized sugars into solution, prolonged attack of the C1 oxidizing LPMO (*Nc*LPMO9F) caused fibrillation of the cellulose nanocrystals as a distinct sign of structural deconstruction. The present results suggest that synergistic interplay between oxidative and hydrolytic enzymes in crystalline cellulose degradation reflects a distinct gain in efficiency of the hydrolytic enzyme as result of the oxidative counterpart’s action. Mechanistically, the efficiency enhancement involves two components. One is an increase in the number of CBH I adsorption sites on the cellulose surface, particularly in areas hardly attacked by the cellulase in the absence of LPMO. The other is enhanced dynamics in elementary steps of CBH I action. The frequency of adsorption and desorption events is increased and the bound cellulases show enhanced processive mobility. Each of these steps is considered to be potentially rate-limiting for hydrolysis^6, 35, 36^. The results also reveal a time scale for the action of adsorbed LPMO substantially longer (min compared to seconds) than that of the processively active CBH I. An important problem, made evident by the current study, is therefore to ensure efficient coupling of oxidative and hydrolytic action over an extended course of cellulose saccharification.

## Methods

### Materials

Unless stated, all chemicals were of the highest purity available from Carl Roth + Co KG (Karlsruhe, Germany).

### Preparation of cellulose nanocrystals

Cellulose nanocrystals were produced according to literature^37^. Whatman^®^ qualitative filter paper (Grade 1; Sigma-Aldrich) was cut into squared pieces of 2 × 2 mm size. About 4 g of cellulose were hydrolyzed in 70 ml of 64 % (w/w) of sulfuric acid for 45 min at 45 °C. The reaction was stopped by 10-fold dilution with deionized water. Repeated centrifugation and washing with deionized water was used to bring the suspension to a final pH of 1.5 or higher. Finally, a colloidal cellulose preparation was obtained by repeated 1 min long sonification (Sonoplus; Bandelin electronic GmbH & Co. KG, Berlin, Germany) of the suspension cooled on ice. Concentration of the solution was determined to be 15 g l^−1^ by weighing of the dry mass. The colloidal cellulose nanocrystal suspension was stored at 4 °C until further use.

### Characterization of cellulose nanocrystals

The crystallinity index (*C*_i_) of the obtained cellulose nanocrystal preparation was calculated to be ≥ 90% using wide angle X-ray scattering^38^. Raman spectroscopy^38^, was used to identify the cellulose allomorph as I_β_ by comparison with corresponding reference spectra from Whatman^®^ qualitative filter paper. Transmission electron microscopy and AFM were used to determine the morphology of the cellulose nanocrystals. The image data were fitted as normal distribution and average values of 127 ± 45 nm in length and 17 ± 6 nm in width were calculated for the more or less rod-shaped cellulose nanocrystals. The detailed experimental setup and staining procedure for Transmission electron microscopy investigations were reported previously^38^. A FT-Raman-based method developed by Zhang and coworkers^39^ was used to assess the amount of sulfate half-ester groups on the surface of the cellulose nanocrystals quantitatively. A calculation based on the normalized areas under the Raman bands between 843 and 825 cm^−1^ indicated a degree of surface substitution (DS_s_) below 0.2 (Supplementary Fig. 6). The data fitting and peak deconvolution were performed using Origin 9 (OriginLab cooperation, Northampton, MA, USA). Raman data were analyzed using LabSpec 6 (Horiba, Tulln an der Donau, Austria).

### Sample preparation for AFM observations

Highly oriented pyrolytic graphite (HOPG) grade I wafers (SPI Supplies, West Chester, PA, USA) were used for fixation of the cellulose nanocrystals^25, 26^. The HOPG wafer surface (10 × 10 mm) was prepared by removing the top graphite layer with adhesive tape followed by immediate incubation with 500 µl of a cellulose nanocrystal suspension diluted to a concentration of 0.6 g l^−1^ with deionized water. Note that concentration and volume of the cellulose nanocrystal suspension were selected to avoid aggregation of nanocrystals on the HOPG wafer. After 10 min of incubation, the HOPG wafer was rinsed with 10 ml of deionized water and dried via CO_2_ spraying. A vacuum chuck was used to mount the HOPG wafer for succeeding AFM observations.

### Enzyme preparations

CBH I was isolated from a cellulase preparation obtained from *T. reesei* SVG17 using a slightly modified ion-exchange protocol as published elsewhere^40^. Purification was done at room temperature. Cellulase was applied to a 6 ml pre-packed Resource Q column (GE Healthcare, Little Chalfont, United Kingdom) equilibrated with 20 mM triethanolamine, pH 7.0. Elution was done with a linear gradient of 0–300 mM NaCl over 10 column volumes. CBH I elutes in a discrete protein peak at about 180 mM salt, clearly separated from other known cellulase activities. Enzyme was gel-filtered to remove salt and stored in 50 mM sodium citrate buffer, pH 5 at 4 °C. Purity of CBH I was confirmed by SDS PAGE, where isolated enzyme migrated as single Coomassie-stained protein band. Specific activity of CBH I assayed with methylumbelliferyl-β-D-cellobioside^41^ was comparable to that of a commercial enzyme preparation (Megazyme, Dublin, Ireland).

Purified preparations of two LPMOs, one C1 (*Nc*LPMO9F) and the other C4 (*Nc*LPMO9C) oxidizing, were obtained through reported procedures^21^. The production of hydrogen peroxide by the purified enzymes was assayed with a fluorimetric assay using L-ascorbic acid as reducing agent as published elsewhere^21^.

The production of oxidized cello-oligosaccharides was measured using cellulose nanocrystals (0.5 g l^−1^) as substrate. Reactions were conducted in 50 mM sodium acetate, pH 5.0, supplemented with 500 µM L-ascorbic acid, in a total reaction volume of 1 ml at 25 °C in Eppendorf tubes sealed with oxygen-permeable Parafilm. Enzyme concentration was 16 µg ml^−1^. The samples were shaken at 400 rpm in an Eppendorf Thermomixer comfort (Eppendorf AG, Hamburg, Germany). The reaction was stopped by adding an equal volume of 100 mM sodium hydroxide to the reaction mixture after 45 min or 90 min, respectively. The samples were centrifuged ( × 10,000 *g*) for 5 min at 8 °C, and the cleared supernatant was assayed for oxidized cello-oligosaccharides as shown in Supplementary Fig. 7.

Aliquots of the LPMOs were stored at −70 °C using a protein concentration of either 8 g l^−1^ (*Nc*LPMO9F) or 55 g l^−1^ (*Nc*LPMO9C) in 20 mM TrisHCl buffer, pH 8. Concentrations of purified enzyme preparations were determined via UV absorbance at 280 nm using molar extinction coefficients calculated from their protein sequence on Uniprot using Protparam (CBH I) or taken from literature (LPMOs)^21^. (ε_CBH I = 86,760 M^−1^ cm^−1^; ε_ *Nc*LPMO9F = 51,130 M^−1^ cm^−1^; ε_ *Nc*LPMO9C = 46,910 M^−1^ cm^−1^)

### AFM observations

AFM observations were carried out using a FastScan Bio Atomic Force Microscope (Bruker AXS, Santa Barbara, CA, USA) operated by a Nanoscope V controller. All experiments were conducted in a small-volume (60 µl) flow cell (Bruker AXS) and FastScan D cantilevers were used in tapping mode with a nominal spring constant and tip radius of 0.3 N m^−1^ and 5 nm, respectively.

Prior to image acquisition the small volume cell covering the HOPG wafer was carefully rinsed with reaction buffer (50 mM sodium acetate, pH 5, supplemented with 500 µM L-ascorbic acid) using an in-house build syringe-driven injection system until the system was devoid of macroscopic air bubbles. The system was allowed to equilibrate for 30 min at 25 °C and multiple reference images of varying size were recorded. Continuous image acquisition was started by the carefully rinsing the cell with 200–250 µl pre-warmed buffer solution containing a C1 oxidizing LPMO (*Nc*LPMO9F) (16 µg ml^−1^) or CBH I (8 µg ml^−1^). Scan rate was either ≤ 3.5 or ~ 40 s per frame for high-speed observations or imaging for multiple cellulose nanocrystals, respectively.

Synergy experiments using CBH I and the C1 oxidizing LPMO were conducted using the same enzyme concentrations as described above at 25 °C. The cellulose nanocrystal preparation fixed on the HOPG wafer was incubated with LPMO for 45 min prior to the addition of CBH I. Subsequently, 200–250 µl pre-warmed buffer solution containing CBH I was added and continuous image acquisition continued as soon as possible.

Experiments with a C4 oxidizing LPMO (*Nc*LPMO9C) were conducted in a similar fashion except for the temperature, which was set to 40 °C and a prolonged equilibration phase of one hour.

Set points and drive amplitudes were selected in order to obtain stable scanning with the lowest energy dissipation possible and adapted if required.

AFM image processing and analysis was performed using Gwyddion 2.31 (released 21 February 2013) and Nanoscope Analysis 1.50 (Build R2.103555, Bruker AXS). All images were plane fitted at 1st order and a median filter was applied unless otherwise stated. Digital movies were constructed using an automated MATLAB routine (developed in Version 7.11.1.866 (R2010b) service pack 1) for drift correction in x, y and z dimension, respectively, and Fiji (ImageJ 1.51 g) (National Institute of Health, USA). Tracking of individual particles was done with the TrackMate v3.4.2 plugin in Fiji.

### Synergy between LPMO and CBH I

Synergy of CBH I and LPMO (*Nc*LPMO9F) was studied in 50 mM sodium acetate, pH 5.0, supplemented with 500 µM L-ascorbic acid, in a total reaction volume of 1 ml at 40 °C in Eppendorf tubes sealed with oxygen-permeable Parafilm. The samples were shaken at 400 rpm in an Eppendorf Thermomixer comfort (Eppendorf AG). Substrate concentration was 1 g l^−1^ of cellulose nanocrystals. The reaction was started by adding a negligible volume of buffer containing CBH I (8 µg ml^−1^) supplemented with or without LPMO (16 µg ml^−1^) as control, respectively. Sampling was performed at suitable time points (2.5, 5, 24, and 60 h). In brief, 150 µl of the well-mixed suspension was withdrawn and mixed with 150 μl of 100 mM sodium hydroxide to stop the reaction. Subsequently, the samples were centrifuged ( × 10,000 g) for 5 min at 8 °C, and the cleared supernatant was subjected to sugar quantification.

### Analytics

Glucose and cellobiose and higher oligosaccharides were analyzed with high performance anion exchange chromatography coupled to pulsed-amperometric detection (HPAEC-PAD) (Dionex BioLC, Thermo Fisher Scientific, Waltham, MA, USA)^38^.

The production of oxidized cellulose-oligosaccharides was monitored with the same HPAEC-PAD system equipped with a CarboPac® PA10 column (4 × 250 mm) and a CarboPac PA10 guard column (4 × 50 mm) at 30 °C. Elution of uncharged saccharides was performed at 0.7 ml min^−1^ using 50 mM sodium hydroxide and 20 mM sodium acetate in the mobile phase for 16 min followed by a sodium acetate gradient. Aldonic acids were eluted by a linear gradient from 40 mM up to 400 mM sodium acetate at a flow of 0.7 ml min^−1^ over 20 min. Afterwards, the column was re-equilibrated for nine minutes with 50 mM sodium hydroxide and 20 mM sodium acetate.

d-Glucose, d-cellobiose and d-gluconic acid were used as authentic standards. Identification or quantification of oxidized products was not pursued.

### Data availability

All data are available from the authors upon reasonable request.

## Electronic supplementary material

Supplementary Information

Peer Review File

Supplementary Movie 1

Supplementary Movie 2

Supplementary Movie 3

Supplementary Movie 4

Supplementary Movie 5

Supplementary Movie 6

Description of Additional Supplementary Files
