# Supplementary material for: Single-molecule study of oxidative enzymatic deconstruction of cellulose
Source: Nat Commun. 2017 Oct 12;8:894. doi: 10.1038/s41467-017-01028-y (PMC5638905; doi:10.1038/s41467-017-01028-y)
Supplement: Supplementary file 9 — Description of Additional Supplementary Files [file 41467_2017_1028_MOESM9_ESM.pdf]

Description of Additional Supplementary Files

File Name: Supplementary Movie 1
Description: Real-time AFM observation of CBH I sliding along a cellulose 3 nanocrystal The enzyme's center is moving along the sidewall and slightly downwards 4 toward the end of the sequence. CBH I is outlined in color and a scale bar is included in the 5 images. The grey scale corresponds to heights between 4 and 10 nm, respectively. Image 6 acquisition rate was 1 frame per second.

File Name: Supplementary Movie 2
Description: Real-time AFM observation of CBH I molecules in the absence 9 of LPMO on the top surface of a cellulose nanocrystal Colored frames indicate adsorbed, 10 stationary (magenta) or moving enzymes (green). The CBH I molecule seen at the 42 second 11 mark was used in Figure 1b for tracking analysis. Scale bar is included in the images. Note 12 that all images were contrast-enhanced to focus on molecules located on the top surface. 13 Image acquisition rate was 0.5 frames per second.

File Name: Supplementary Movie 3
Description: Real-time observation of LPMO molecules adsorbing to and 16 desorbing from cellulose nanocrystals Enzymes outlined in color (sidewall: green; top 17 surface: magenta) were present in the previous image. Vacated positions are indicated with a 18 ‘×’ mark. Scale bar is included in the images. The grey scale corresponds to heights between 19 0 and 20 nm

File Name: Supplementary Movie 4
Description: Real-time observation of LPMO molecules adsorbing to and 22 desorbing from multiple cellulose nanocrystals Note that microscopic air bubbles (outlined 23 in blue) were present throughout the whole experiment. Color coding of enzymes is as 24 described for supplementary movie 3. Scale bar is included in the images. The grey scale 25 corresponds to heights between 0 and 20 nm.

File Name: Supplementary Movie 5
Description: Real-time AFM observation of effect of LPMO preincubation 28 on adsorption of CBH I to cellulose nanocrystals Adsorbed enzymes are outlined in color. 29 Scale bar is included and all images were contrast enhanced to focus on molecules located on 30 the top surface.

File Name: Supplementary Movie 6
Description: High-speed AFM observation of action of CBH I on cellulose 33 nanocrystals in the presence of LPMO Clusters of enzymes were labeled with turquoise 34 frames and isolated enzymes originating from clusters were highlighted in yellow. Single 35 enzymes clearly isolated on the surface, labeled magenta (immobile) or green (moving), were 36 used for tracking analysis. Note:

labeling in color was used only to highlight exemplary 37 clusters of enzymes or individual enzymes. It was not employed throughout the entire video. 38 Image acquisition rate was 0.2 frames per second. Note that all images were contrast 39 enhanced for clarity by setting gamma to 0.1 using CorelDrawX8. Scale bar is included in the 40 images.
