# Supplementary material for: Single-molecule study of oxidative enzymatic deconstruction of cellulose
Source: Nat Commun. 2017 Oct 12;8:894. doi: 10.1038/s41467-017-01028-y (PMC5638905; doi:10.1038/s41467-017-01028-y)
Supplement: Supplementary file 1 — Supplementary Information [file 41467_2017_1028_MOESM1_ESM.pdf]

Reviewer #1 (Remarks to the Author):

This paper reports the AFM imaging of cellulose-degrading enzymes on crystalline cellulose substrates. One of the cellulose-degrading enzymes classes is an LPMO, the recently discovered enzymes which are know to oxidatively cleave polysaccharide chains.

The paper's main findings are that LPMOs bind to the hydrophobic surfaces of cellulose where they have long residence times, much greater than other processive cellulases. The paper also shows the clear defibrillation of the cellulose under LPMO action.

The paper is quite well written, with clear explanations of the data. The conclusions are well supported by the AFM data. Very few amendments are required (see below).

Given the current important context of LPMOs in biomass degradation, any insight into their mode of action is a significant addition to the literature. As such, I am happy to recommend publication in Nat Commun.

Suggested changes:

I think it would be helpful for the authors to cite the original LPMO papers which announced the discovery of LPMOs (Science, 2010, 330(8), pg 219) and their copper active sites (PNAS, 2011, 15079).

I would not describe the active site of LPMO as homonuclear, but as mononuclear.

Reviewer #2 (Remarks to the Author):

The manuscript from Eibinger et al. employs real-time AFM to demonstrate that LPMOs bind to the same surfaces of cellulose as cellobiohydrolases. Moreover, the study shows that LPMO action significantly improves the adsorption and desorption of cellobiohydrolases on cellulose. Interestingly, the study also shows that the LPMOs studied here do not appreciably diffuse on cellulose.

I enjoyed reading this paper, and think that it offers new insights into the (lack of) mobility of LPMOs on the cellulose surface, which is a bit surprising, and the direct observation of LPMO action to create more adsorption and desorption sites for Cel7A (CBH I) - a key finding often speculated on, especially given the recent discoveries from Westh, Valjamae, and Igarashi that desorption of Cel7A is likely rate-limiting in the absence of accessory enzymes (such as endoglucanases and, as shown here, LPMOs).

Is there a way for the authors to know what the "side walls" of the cellulose nanocrystals are in terms of cellulose crystal face? (I have no idea how to do this, but wonder if the authors do). The speculation regarding this being the hydrophobic face makes sense given the information known about how Cel7A works, but it would be of interest to know if the authors had a way to characterize this in the AFM measurements. Does the preferred orientation of the cellulose on the wafer relative to the wafer surface chemistry give any clues about this?

Although I don't think that the authors need to do this for this particular study, I think it would add a lot of impact to the work conducted here to use an endoglucanase in concert with Cel7A to do the same types of analyses. Are the results in Figure 3d also observed in that case? That would be very interesting.

Is the adsorption of the CBM-less and CBM-containing LPMO similar in terms of residence time? Which LPMO is shown in the figures in the main text?

What does the concept of "upward, downward or horizontal" mean in terms of how a crystal is oriented and an enzyme moves on it? This is a bit unclear and could use clarification.

The authors should read (if they have not already) and cite the newly posted manuscript from the Eijsink group showing that hydrogen peroxide is the co-factor of LPMOs, not oxygen. This is currently on bioRxiv.

Reviewer #3 (Remarks to the Author):

See attachment

Response letter to referee reports on manuscript NCOMMS-17-04158-T “Single molecule study of oxidative enzymatic deconstruction of cellulose” by Manuel Eibinger, Jürgen Sattelkow, Thomas Ganner, Harald Plank, and Bernd Nidetzky

We wish to thank the reviewers for their comments and the constructive critique made. A revised version of the manuscript was prepared in which all points were considered. In our view, and we hope reviewers will agree, we were able to address all points properly and exhaustively. New evidence was obtained in the course of revision (enzyme height measurements; high-speed AFM analysis of synergy between LPMO and CBH I) and the original AFM data were analyzed rigorously according to suggestions made by reviewer #3. Below we provide response to the reviewers' suggestions and points of criticism in a point-by-point fashion. We hope that having been revised carefully along lines suggested by the reviewers, the manuscript will now be considered to be acceptable for publication.

Reviewer #1

This paper reports the AFM imaging of cellulose-degrading enzymes on crystalline cellulose substrates. One of the cellulose-degrading enzymes classes is an LPMO, the recently discovered enzymes which are know to oxidatively cleave polysaccharide chains.

The paper's main findings are that LPMOs bind to the hydrophobic surfaces of cellulose where they have long residence times, much greater than other processive cellulases. The paper also shows the clear defibrillation of the cellulose under LPMO action.

The paper is quite well written, with clear explanations of the data. The conclusions are well supported by the AFM data. Very few amendments are required (see below).

Given the current important context of LPMOs in biomass degradation, any insight into their mode of action is a significant addition to the literature. As such, I am happy to recommend publication in Nat Commun.

Comment 1.1: I think it would be helpful for the authors to cite the original LPMO papers which announced the discovery of LPMOs (Science, 2010, 330(8), pg 219) and their copper active sites (PNAS, 2011, 15079).

Response 1.1: The seminal work of Vaaje-Kolstad et al., published in Science and reporting the discovery of LPMO activity on recalcitrant polysaccharides, was already cited at a prominent position in the introduction of the original manuscript (Ref. 8). However, we agree that the work of Quinlan at al., characterizing the structure of LPMO with a detailed

analysis of the copper active site, deserves just the same attention, and it is therefore included at a suitable position in the introduction.

Comment 1.2: I would not describe the active site of LPMO as homonuclear, but as mononuclear.

Response 1.2: We agree with the reviewer and therefore adapted the description accordingly.

Reviewer #2

The manuscript from Eibinger et al. employs real-time AFM to demonstrate that LPMOs bind to the same surfaces of cellulose as cellobiohydrolases. Moreover, the study shows that LPMO action significantly improves the adsorption and desorption of cellobiohydrolases on cellulose. Interestingly, the study also shows that the LPMOs studied here do not appreciably diffuse on cellulose.

I enjoyed reading this paper, and think that it offers new insights into the (lack of) mobility of LPMOs on the cellulose surface, which is a bit surprising, and the direct observation of LPMO action to create more adsorption and desorption sites for Cel7A (CBH I) - a key finding often speculated on, especially given the recent discoveries from Westh, Valjamae, and Igarashi that desorption of Cel7A is likely rate-limiting in the absence of accessory enzymes (such as endoglucanases and, as shown here, LPMOs).

Comment 2.1: Is there a way for the authors to know what the "side walls" of the cellulose nanocrystals are in terms of cellulose crystal face? (I have no idea how to do this, but wonder if the authors do). The speculation regarding this being the hydrophobic face makes sense given the information known about how Cel7A works, but it would be of interest to know if the authors had a way to characterize this in the AFM measurements. Does the preferred orientation of the cellulose on the wafer relative to the wafer surface chemistry give any clues about this?

Response 2.1: We thank the reviewer for this important comment. We agree that the location and orientation of the hydrophobic faces of the cellulose nanocrystal is of considerable interest. However, we must be clear stating that AFM can't be readily used to clearly localize these faces in the substrate. In previous AFM studies from the seminal paper of Igarashi and colleagues, based on the geometry of slightly different cellulose crystals (derived from bacterial origin), it was suggested that the crystal would orient on the surface of

the grid such that the hydrophobic lanes are located at the side walls. In our case, crystals are of cellulose allomorph (I_β) and according to their aspect ratio, they resemble in morphology the cellulose nanocrystals obtained from acid hydrolysis of tunicates. Now, with the plausible assumption of their wide side facing downwards, the nanocrystals used in our study should expose their hydrophobic faces also on the side walls. Orientation in the way just described would agree with the evidence that enzymes were predominately adsorbing to, and were acting on, the side walls. However, we must emphasize again that the proposed orientation of the nanocrystal is supported only by indirect evidenec. The statement in the main text addressing this topic was slightly expanded. We feel that interpretation must be made with due caution at this stage.

Comment 2.2: Although I don't think that the authors need to do this for this particular study, I think it would add a lot of impact to the work conducted here to use an endoglucanase in concert with Cel7A to do the same types of analyses. Are the results in Figure 3d also observed in that case? That would be very interesting.

Response 2.2: We agree that such a setup (using different cellulases in combination with LPMO) would be interesting, and it constitutes research we might pursue in the future. We know from previous experience, using a different type of cellulose however, that crystalline areas of cellulose were not attacked by EG to an extent visible in AFM analysis. Synergy between LPMO and hydrolytic cellulases at the level of AFM visualization was previously observed in the main for CBH I (Eibinger et al., 2014, J. Biol. Chem. 289, 35929). Therefore, as far as synergy between LPMO and hydrolytic enzymes was concerned, it seemed to make sense that we prioritize experiments with CBH I. Analysis of all basic types of cellulase, that is, EG but also CBH II, would have been too broad a scope to be addressed in the current study. In this context, therefore, we also would like to emphasize that for a large part, the current study is a single molecule analysis of LPMO in its *individual* interaction with cellulose. Since CBH I, in addition to showing synergy with LPMO, exhibits such a characteristic behavior on the cellulose surface, that is, it moves unidirectionally in consequence of processive polysaccharide chain cleavage, it seemed appropriate and useful to apply just this enzyme as a reference to the analysis of the LPMO.

Comment 2.3: Is the adsorption of the CBM-less and CBM-containing LPMO similar in terms of residence time? Which LPMO is shown in the figures in the main text?

Response 2.3: We thank the reviewer for drawing attention to this important point which must not be confused. Most of the data reported in the manuscript are from the analysis of the C1 oxidizing LPMO that does not contain a CBM. Text and legend to figures were revised to make clear which type of LPMO was used to obtain the results shown. As for the C4 oxidizing LPMO that is equipped with a CBM, we were primarily interested in potential differences to the C1 oxidizing LPMO as to the sites of adsorption and a possible mobility/movement on the cellulose surface. The two enzymes did not differ in these two respects. Most notably, both enzymes appeared essentially immobile on the cellulose surface. In the case of the C4 oxidizing LPMO, the amount of enzyme molecules studied was not sufficient to determine a residence time on the surface. We agree that a quantitative structure-function analysis in which the presence of a CBM is related to residence time on cellulose could be interesting, but this would require a different study design and goes beyond what the current research aimed at clarifying.

Comment 2.4: What does the concept of "upward, downward or horizontal" mean in terms of how a crystal is oriented and an enzyme moves on it? This is a bit unclear and could use clarification.

Response 2.4: We agree with the reviewer that the description used was not entirely clear. We edited Figure 1 to include a new scheme that shows an idealized cellulose nanocrystal attached to the AFM support. The figure also clarifies the terminology used.

Comment 2.5: The authors should read (if they have not already) and cite the newly posted manuscript from the Eijsink group showing that hydrogen peroxide is the co-factor of LPMOs, not oxygen. This is currently on bioRxiv.

Response 2.5: We agree with the reviewer and give citation of this potentially highly significant paper. We are sure that the paper will trigger interesting discussion about the use of co-substrate in the LPMO reaction. However, our study is certainly not the right place to elaborate on this important mechanistic feature of LPMO in more detail. We have no evidence in taking sides in this debate. However, considering proposal from the paper of Eijsink and colleagues that H_2O_2 is used by LPMO rather than O_2, we feel it is necessary to emphasize that in the reaction set-up used in our study (O_2 present, H_2O_2 lacking), LPMO showed clear activity. Besides seeing fibrillation of cellulose crystals as evidence of structural disintegration of cellulose, we were able to also observe the release of oxidized soluble sugars. The sugar release was significant in the timespan of our AFM experiments. These

results clearly suggest that the LPMO was active under the conditions used. It is possible that "optimum reaction conditions" for LPMO need to be revised in the light of the evidence from Eijsink et al., but it is also fact that without hydrogen peroxide added, the enzyme also showed substantial activity. We therefore think that while suitable credit should be given to Eijsink's study by citation, their results in no way invalidate the experimental set-up used in the current work.

Reviewer #3

Eibinger et al applied time-resolved AFM to observation of decomposition process of crystalline cellulose with LPMO and CBHI. They found that LPMO is bound to cellulose surface with a longer dwell of 0.6 min compared to the CBHI and then induces fibrillation of the cellulose nanocrystal. Also they addressed the molecular mechanism of synergetic effect between LPMO and CBHI in which the adsorption of CBHI onto the cellulose surface is strongly increased by the presence of LPMO. Effective degradation of cellulose is very important in terms of biomass conversion and therefore understanding the molecular mechanisms of the enzymatic activity and its enhancement due to oxidative enzymes is a key for practical use of plant biomass. From this point of view, the conclusions based on the single-molecule observation sound quite interesting and would provide novel insight into molecular mechanism of cellulose degradation by CBHs. However the overall quality of the AFM images presented in the manuscript is quite poor and quantitative analysis is insufficient to support the conclusion. Hence I hesitate to recommend the manuscript to be accepted for publication in Nature Communications. Here I describe specific points of my concerns raised.

Comment 3.1: Fig.1a (Supplementary Movie 1): Although the authors insist that the AFM movie shows unidirectional movement of the CBHI along the cellulose nanocrystal from the movie 1, I can not agree with that. I can recognize the movement of the white spot between only two frames (9s and 12 s) but it is hard to say the unidirectional processive movement only from this data. More number of enzymes moving in the same direction should be clearly shown. Furthermore, size of the white spot that the authors consider the CBHI seems too large to the CBHI and the shape is a bit strange. Does the spot really correspond to a single enzyme? How about the possibility of aggregate?

Response 3.1: We agree with the reviewer that the selected image sequence should have been better chosen to show the processive movement of a single enzyme. To obtain data with improved temporal resolution, we performed additional experiments with an increased

frame recording rate, reorganized Figure 1 and provided two new movies (supplementary movies 1 and 2). More specifically, Figure 1b contains a new continuous image sequence and a short trajectory profile over three frames omitting post- and pre-frames of the crystal without enzyme. The frame recording rate was 0.5 s^{-1} and the images were taken from the supplementary movie 2. Movie S1 was replaced with a different continuous sequence of seven images with a frame recording rate of 1 s^{-1}. The Figure caption was updated accordingly. We think that the evidence now provided is sufficient to demonstrate the movement of the enzyme and to use the results as a frame of reference for the analysis of LPMO. We are fully aware that a time resolution higher than the one usable here would have improved on the tracking of individual CBH I molecules in movement. However, our priority was LPMO.

Comment 3.2: Fig.1b (supplementary Movie 2): When the LPMO adsorbs to the side wall of the cellulose crystal, it would be hard to judge whether the LPMO adsorbs the crystal or the HOPG substrate. Actually some LPMOs look to adsorb onto the substrate but not the cellulose because one can see a gap between the spot and cellulose fibril. The authors count even those molecule as be bound to the cellulose. How do the authors judge whether the LPMO adsorb to the cellulose? What is the criteria?

Response 3.2: We thank the reviewer for the careful assessment of our AFM data and this important comment. In fact, a completely clear-cut separation of enzymes attached just to a crystallite, and not to the surrounding graphite, is quite challenging. However, based on a detailed and rigorously performed analysis, we were able to decrease the percentage of enzyme molecules, for which it was not completely clear whether they were bound just to cellulose or to cellulose and graphite at the same time, to 15% or less of the total number. We think this level of accuracy to be acceptable. We included a short paragraph in the manuscript clarifying this point. The analysis was performed in multiple steps, thereby ensuring correct localization of the enzymes. First, a visual inspection of the raw and filtered height and amplitude data was carried out. Candidate enzymes to be bound to cellulose were identified thus. Secondly, height profiles of candidate enzymes were obtained along the edge of the nanocrystals (see Figure R1). Key criterion to recognize an enzyme as bound to the sidewall of the nanocrystal was the height difference of at least 2.5 nm between the highest point of the LPMO and the graphite support lying underneath.

The key criterion was carefully chosen based on our height data obtained for LPMO (supplementary Figure 1a, μ = 1.5 nm; σ = 0.7 nm). The fraction of potentially wrongly

adsorbed LPMOs with an apparent height of more than 2.5 nm was 11% (supplementary Figure 1).

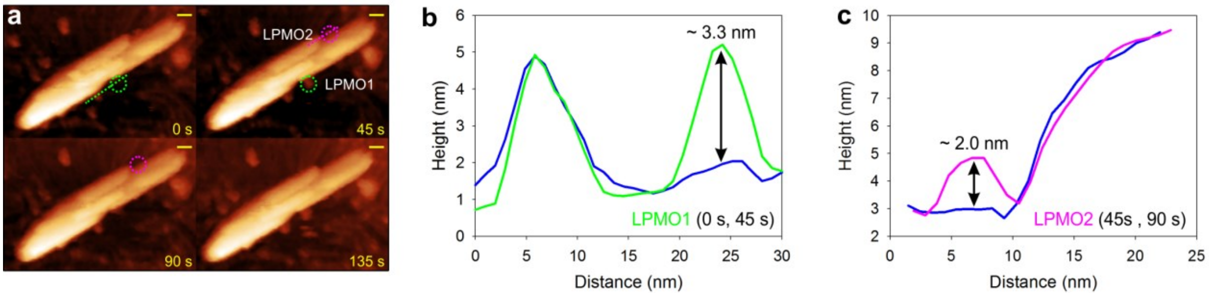

Referee Figure 1. Analysis of height profiles to distinguish LPMO molecules adsorbed solely to the sidewall of cellulose from other LPMO molecules that are bound to the graphite surface also. (a) The height profiles are from the data in Figure 1c and were obtained along the dashed lines. The crystal/graphite support background is drawn in blue and height profiles including LPMOs are colored green (LPMO1, panel b) and magenta (LPMO2, panel c). (b) LPMO1 and (c) LPMO2 were selected as potential candidates for adsorption based on visual inspection. LPMO1 was ~3.3 nm in height and hence recognized as bound to the sidewall of the nanocrystal. LPMO2 failed to satisfy the height criterion (height ~2.0 nm) and was subsequently not recognized as bound to the crystal. Please note that the obtained height profile of LPMO2 clearly suggests that the lateral resolution was sufficient at the crystal/graphite support interface to allow for rigorous analysis. All scale bars are 10 nm.

Comment 3.3: p.5, “Crowding of LPMO and CBHI…”: What does “crowding” mean? In Fig. 1d, I can not see any crowding of molecule but only three molecules which are separately bound to the crystal.

Response 3.3: With "crowding" we meant that enzyme come into close(r) proximity on the cellulose surface as conditions are changed. We agree that the term was probably misleading and re-phrased the sentence by using the term "co-localization".

Comment 3.4: Fig. 1d: The authors distinguish CBHI from LPMO by their heights. The authors should show that the CBHI and LPMO are observed as different heights (size) on the flat surface such as HOPG surface and the height difference is consistent with the result of Fig.1d. Also it should be confirmed by changing the concentrations of molecules and then see change in the number of observed molecules with different size.

Response 3.4: We agree with the reviewer and performed additional experiments as suggested. We analyzed the maximum height of multiple hundreds of isolated enzymes located on the graphite support and show the results in the histograms provided in Figure S2. Histograms were fitted according to a Gaussian distribution and the obtained average maximum height for CBH I and LPMO are 2.8 nm and 1.5 nm, respectively. Interestingly, we also observed two distinct maxima for CBH I indicating that this approach was capable of resolving the two-domain structure of the enzymes. An example is provided in supplementary Fig. 1b. Moreover, we added CBH I to adsorbed LPMOs (shown supplementary Fig. 1c) and the height profile collected from the sample showed the appearance of a shoulder in the histogram. The newly obtained histogram was fitted according to a Gaussian distribution and two distinct maxima were found. Maxima were located at 1.8 and 3.2 nm, respectively, which was in good agreement with height values for the CBH I catalytic core domain and the LPMO determined in previous experiments that were reported in the original version of the manuscript. Please note that the additional analysis presented in the revised manuscript reinforces our claim that CBH I was roughly twice as high as LPMO in the AFM data. Moreover, it supported the rigorous analysis (see response to comment 3.2) through which we determined that most of the LPMOs were bound to the sidewall of the crystallites (without being bound to surface of the graphite at the same time).

Comment 3.5: Fig. 2a (supplementary Movie 3): Again some LPMO molecules are obviously adsorbed onto the HOPG but not on the cellulose. If the authors make the histogram (Fig .2b) from this movie, events are probably overrated. The molecules which clearly adsorb onto the crystal should be evaluated. Thus it is hard for me to trust the quantification of the residence time.

Response 3.5: We agree with the reviewer and performed a new, very rigorous analysis of the time-resolved AFM sequences as regards the distinction between enzymes that were adsorbed to cellulose unambiguously and other enzymes that may have been bound to cellulose and graphite at the same time. The procedure outlined in the responses to comments 3.2 and 3.4 was used. Movie S2, Movie S3 (now S3 and S4), Figure 1c-d and Figure 2a-c were updated. We would like to emphasize that while the shown movies were initially 3x3 median-filtered for easier viewing, the quantitative analysis of adsorbed LPMOs was done using the raw AFM data without any filtering applied.

Comment 3.6: Fig. 3a (supplementary Figure 2): Some movies of fibrillation process shown as figures should be supplemented to see more details. The size of LPMO in the supplementary Figure 2a looks larger than that shown in Fig. 1b. What is the reason? Is this actually LPMO? Also did the authors observe more LPMO on the same crystal and the fibrillation at many regions ta the same time?

Response 3.6: Fibrillation of cellulose was observed only after extended incubation times that were not suitable for "recording movies" in a fully continuous time-resolved analysis. Multiple fibrillation events in a single cellulose crystal were not observed. Fibrillation generally was a process seen only in a limited number of crystals (10 - 20%) and was therefore considered as a late stage effect of LPMO activity. Although fibrillation was definitely related causally to the presence of LPMO, that is, it was an event never seen in the absence of the enzyme, we cannot rigorously exclude the possibility that interaction with the AFM tip facilitated the full detachment of the (already loosened) cellulose fibril from the crystal so as to become visible eventually in the AFM scan. A minor size difference between the LPMO observed in supplementary Fig. 2a and Fig. 1b is attributed to tip convolution effects and additional broadening of the feature due to median filtering.

Comment 3.7: Fig. 3b (supplementary Movie 4): While most of LPMO adsorbed to the side of the crystal in Fig. 1b, CBHI adsorbs on the top surface of the crystal after the treatment with LPMO. Why is the binding of CBHI to the crystal enhanced even though LPMO preferentially binds to the side? Does CBHI adsorb on only the site where LPMO attack? If so, why is the number of LPMO adsorbed on the crystal is obviously fewer than CBHI as shown the movie 4?

Response 3.7: We thank the reviewer for this important comment. Although it is not readily visible in Figure 3b due to a z-scaling that focuses on the top surface (4 nm offset), the amount of CBH I molecules attached to the sidewall is actually increased to a significant extent. To clarify this situation, we added a new supplementary Figure 4 showing the same image sequence as depicted in Fig. 3b with an adapted z-scaling to focus on the enzymes located at the sidewall. Additionally, reference images from cellulose nanocrystals incubated with either LPMO or CBH I are provided for visual comparison. However, as we say in the manuscript, quantification was very difficult, impossible even over the whole area of the crystal, due to the high abundance of enzymes adsorbed in close proximity. Supplementary Figure 4 shows this effect which we originally referred to as "crowding".

Comment 3.8: Supplementary Movie 5 and Figure 3: I can't see clear processive movements of CBHI. I wonder how the author estimated the maximum velocities using the ambiguous movies. Also it seems that the number of molecules analyzed for the histograms in supplementary Figure 3 is not enough for quantitative analysis and therefore the distributions of the maximum velocity is not reliable. The number of molecules observed should be increased to have more rigid analysis. Also the ratio of the moving CBHI to all molecules observed should be indicated.

Response 3.8: We agree that with the description given in the original version of the manuscript these results were not clear. We therefore replaced them with a different, clear image sequence. Supplementary Movie 6 (previously supplementary movie 5) shows two effects of LPMO on the action of CBH I. First, when LPMO was present, an increased abundance of CBH molecules was observed on the top surface of the cellulose nanocrystals. Secondly, a greater number of CBH I molecules showed movement on the surface (e.g. top-left corner, 1 min 25 seconds mark). An immediate conclusion from the results in supplementary Movie 5 was that LPMO enhances the adsorption of CBH I (at the top surface of the cellulose at least) and that it also increases the apparent mobility of the CBH I molecules on that surface. This led us to say that LPMO activity appears to invigorate the action of CBH I. At a qualitative level, this statement is supported very clearly by comparing the results of supplementary movie 6 with the results of supplementary movie 2. Now for the velocity profile, which is shown in supplementary Figure 5, please note that this was not calculated solely on the AFM sequences depicted in MovieS5. The analysis was done over ~120 individual frames by using automated particle tracking with the TrackMate v3.4.2 plugin in Fiji. Maximal displacement of an enzyme between two frames was set to 35 nm (velocity of CBH I: 7 nm/s; time resolution: 0.2 fps) and splitting of individual tracks was not allowed. Thus individual tracks for well over 60 enzymes were obtained. Reference data was obtained from a different experiment using CBH I at the same concentration as in supplementary Movie S6 but in the absence of LPMO. Two individual nanocrystals were analyzed over ~300 frames in total and the time resolution was 0.3 fps. Analysis parameters were the same as for LPMO/CBH I but the maximal displacement was changed to 25 nm to account for the increased frame recording rate. The legend to supplementary Figure 5 was revised to indicate more clearly the data analyzed and the procedures used.

We are aware of the technical limitations of the experiment and the analysis performed. There are restrictions as regards the frame rate, and the number of enzyme molecules analyzed is at the lower end demanded by statistics. However, we must recall that the experiment did not try

to measure the velocity of CBH I with the highest possible precision but rather aimed at comparing, relative to each other, the velocity distributions of CBH I in the absence and presence of LPMO. We retain that for a comparative analysis such as this, the data are useful and significant, revealing an overall shift of the velocity profile to higher values when LPMO is present. We therefore think that the data can, and should, be shown in the Supporting Information.

In the analysis of CBH I moving on the top surface of the cellulose, the portion of enzymes in processive motion, independent of the presence or absence of LPMO, was approximately 30%. A statement indicating this was included in the results part of the manuscript. However, we have to emphasize that the fraction of mobile enzymes is presumably underrated in the presence of LPMO due to the massive accumulation of enzymes, which we referred to previously as "crowding" (shown in supplementary Movie S6), thus complicating the tracking of individual particles. The absolute amount of CBH I molecules in motion is significantly increased as shown by the simple comparison of supplementary Movies 2 and 6.

Just to note, immobile CBH I enzymes on the cellulose surface represent an already reported phenomenon which can most likely be attributed to non-productive adsorption of the enzymes[1].

References

1. Nakamura, A. *et al.* Single-molecule Imaging Analysis of Binding, Processive Movement, and Dissociation of Cellobiohydrolase Trichoderma reesei Cel6A and Its Domains on Crystalline Cellulose. *J. Biol. Chem.* **291,** 22404–22413 (2016).

Reviewer #3 (Remarks to the Author):

I have read the authors' rebuttal letter and, checked the responses and corresponding improved figures. The author sincerely gave answers to my questions and image quality is significantly improved. I am satisfied with that and thus agree to accept the manuscript for publication in Nat. Communications.
