# Supplementary material for: Single-molecule study of oxidative enzymatic deconstruction of cellulose
Source: Nat Commun. 2017 Oct 12;8:894. doi: 10.1038/s41467-017-01028-y (PMC5638905; doi:10.1038/s41467-017-01028-y)
Supplement: Supplementary file 2 — Peer Review File [file 41467_2017_1028_MOESM2_ESM.pdf]

Supplementary Figures

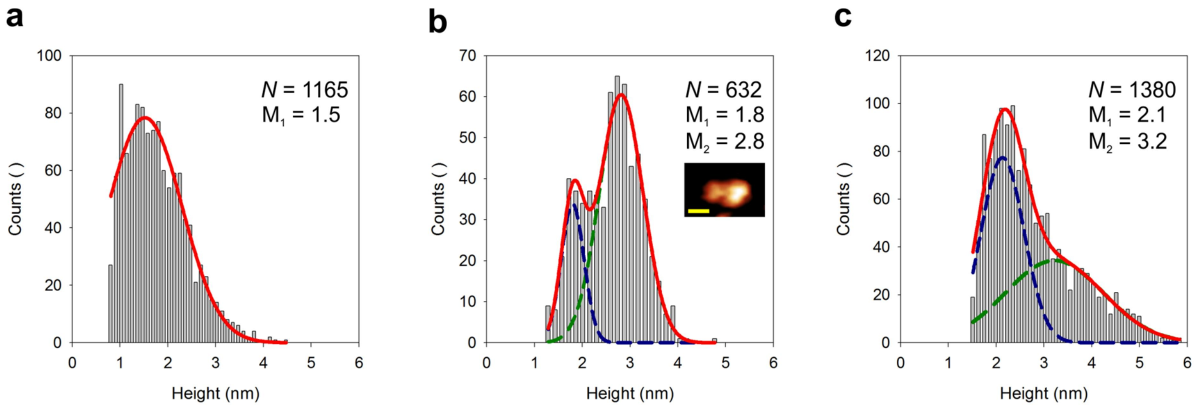

Supplementary Figure 1: AFM observations of enzymes adsorbed on graphite grid Height distributions of LPMO (a), CBH I (b) and a combination of both (c) were obtained through single molecule analysis. Gaussian distributions were fitted to all data sets using an automated MATLAB routine. A unimodal distribution with a discrete maximum was found from the analysis of LPMO while a bimodal distribution was obtained from the analysis of CBH I alone and the mixture of LPMO and CBH I. The individual distributions from the biomodal distribution are indicated with dashed lines. Positions of the maxima (M_1, M_2) and sample size (*N*) are indicated in the histograms. Note that the achieved resolution was sufficient to visualize the two-domain structure (see Payne et al.[1]) of resting CBH I molecules (inset of panel b). The smaller cellulose binding module (left domain) and the larger catalytic core (right domain) of the shown CBH I were 1.7 and 3.1 nm in height, respectively, fully consistent with the sizes of the protein domains known from their atomic structures. The two populations revealed for the combination of LPMO and CBH I are most likely an overlay of LPMO/CBM and the catalytic core of CBH I. Scale bar is 5 nm.

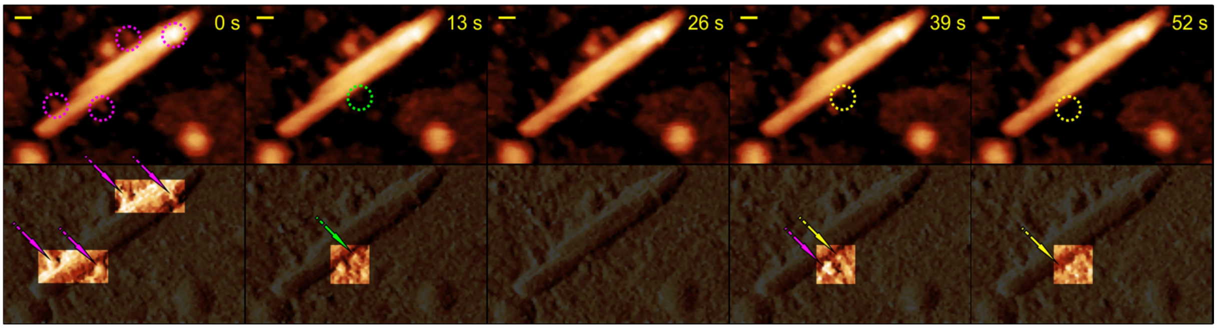

Supplementary Figure 2: Real-time observation of C4 oxidizing LPMO on cellulose Colored frames indicate enzymes present initially (magenta), adsorbing (green) or desorbing (vacated position; yellow) in the depicted timespan. Amplitude images of the corresponding time points are shown in the bottom panel. LPMO molecules are indicated by arrows using the same color code as described above. All scale bars are 10 nm.

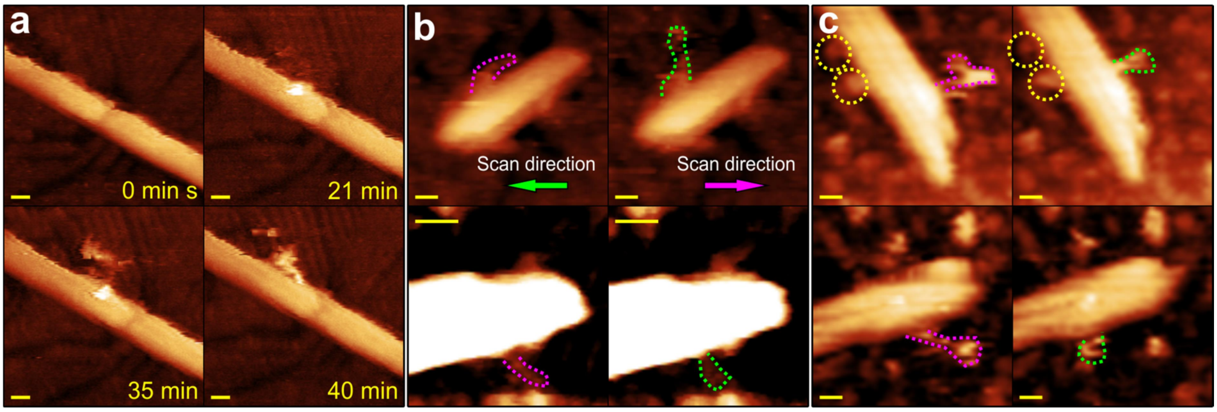

Supplementary Figure 3: Real-time observations of effect of LPMO on structural deconstruction of cellulose nanocrystals (a) Real-time observation of partial detachment of cellulose fibrils from nanocrystals as result of LPMO (*Nc*LPMO9F) activity. An LPMO molecule adsorbs to an intact crystal and a fibril loosened from the crystalline material becomes visible contemporaneously. The frame-recording rate was 1 min^{-1} to provide high-resolution images. (b) By comparing forward (trace; magenta) and backward direction (retrace; green) of AFM line scans, the loosened parts of the fibrils are shown to be often moveable by the AFM tip. The fibril part associated with the cellulose nanocrystal, by contrast, was not moveable. (c) Similar AFM observations concerning fibrillation were made using a C4 oxidizing LPMO (*Nc*LPMO9C) at elevated temperature (40°C). Note that intact LPMO molecules (yellow frames) have a similar shape and position in trace and retrace unlike detached fibers. All scale bars are 10 nm.

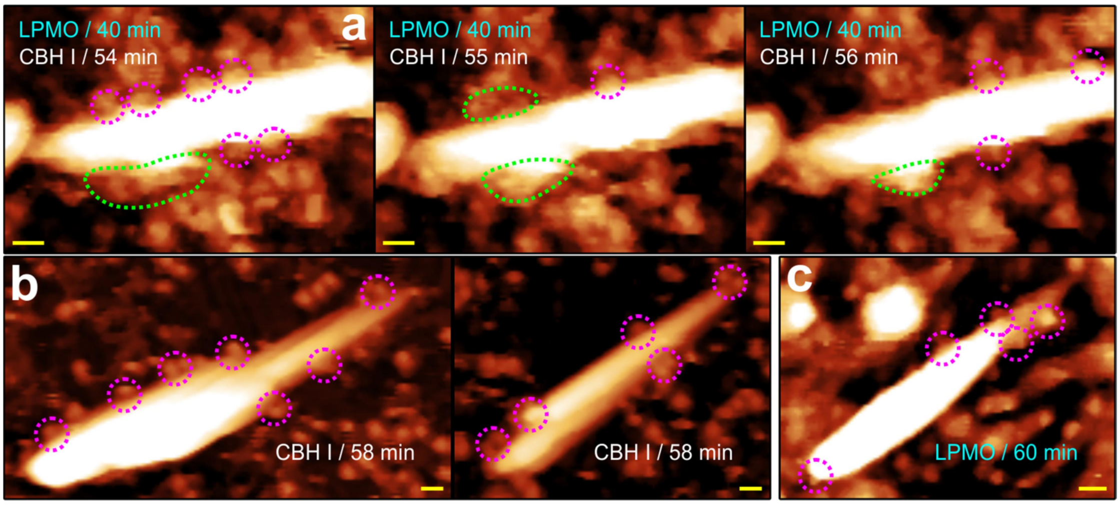

Supplementary Figure 4: Effect of LPMO on enzyme adsorption to the sidewalls of cellulose nanocrystals The experiment used pre-incubation of the nanocrytsals with LPMO for 40 min. CBH I was added afterwards. (a) The image sequence is the same as shown as Figure 3b (except the first panel, pre-incubation with LPMO), however, with an adapted z-scale (1-8 nm) to focus on enzymes adsorbed to the sidewalls of the nanocrystal. Although isolated enzymes were visible (magenta circle), analysis of single molecules for exact quantification was not possible due to the accumulation (crowding) and partial overlapping of multiple enzymes (green frame). However, the visual comparison with reference images obtained from cellulose nanocrystal incubated with CBH I alone (b) or LPMO alone (c) showed clearly that the amount of adsorbed enzymes (magenta circles, independent of enzyme type or position) was increased under conditions when both enzymes were present. All scale bars are 10 nm.

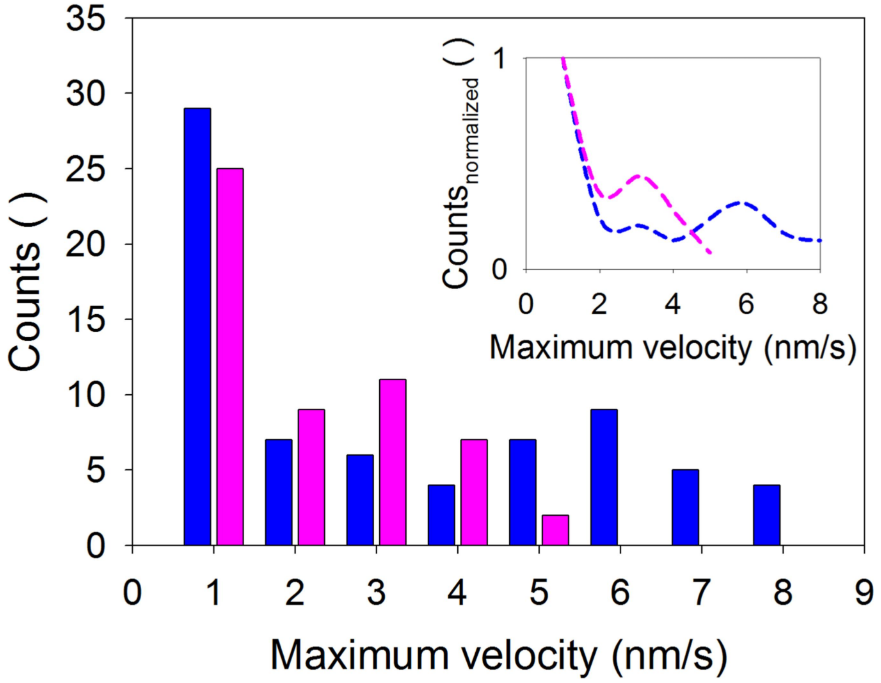

Supplementary Figure 5: Effect of LPMO on CBH I moving on the cellulose surface The distribution of the maximum velocity of CBH I molecules located on the top surface of a nanocrystal in the presence (blue bars) or absence of a C1 oxidizing LPMO (magenta bars) is shown. The apparent maximum velocity of moving CBH I molecules in the presence of LPMO was increased about 1.5-fold as compared to CBH I acting without LPMO. Molecules with an apparent maximum velocity of 1 nm s^{-1} or less were considered as not moving. Analysis of CBH I in the presence of LPMO was based on experiments with a frame-recording rate of 0.2 s^{-1}. A short sequence of the frames used to calculate the apparent maximum velocity is shown in supplementary Movie 5. CBH I in the absence of LPMO was analyzed with a frame-recording rate of 0.3 s^{-1} (exemplified in Figure 1b). Tracking of individual particles and calculation of the maximum velocity were done with the TrackMate v3.4.2 plugin in Fiji. Splitting of individual tracks was not allowed and the amount of analyzed molecules was ≥ 55 for each scenario. The maximal displacement of an enzyme between two frames was calculated based on the reported velocity of CBH I (7 nm s^{-1})[2] and the achieved time resolution. The inset shows the same distribution as a normalized line graphic for easier viewing.

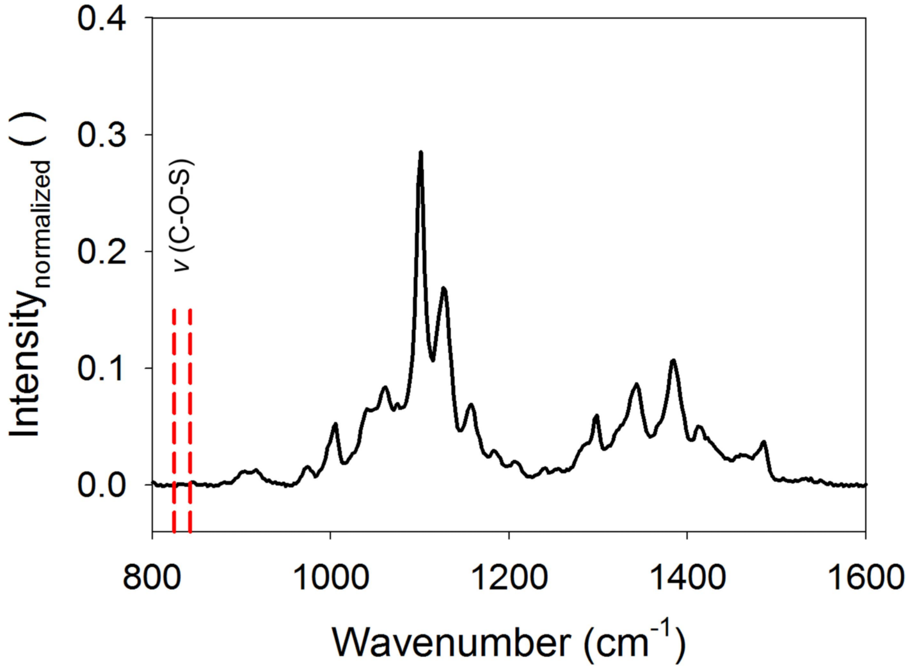

Supplementary Figure 6: Raman spectroscopy of cellulose nanocrystals The degree of surface substitution (DS_s) can be calculated based on the normalized areas under the Raman bands between wavenumbers 843 cm^{-1} and 825 cm^{-1}, which correspond to the C–O–S stretching vibration, *ν*(C-O-S). No clear bands were observed in that region, implying a DS_s below the detection limit (~ 0.2) of the method. Baseline correction and a vector-normalization were done using LabSpec 6.

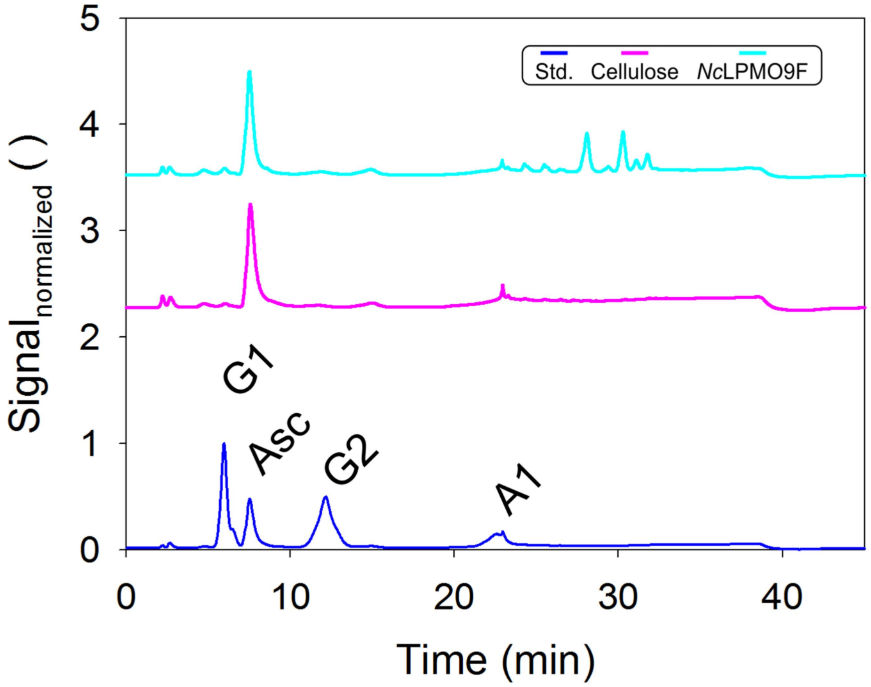

Supplementary Figure 7: HPAEC-PAD chromatogram of LPMO incubated with cellulose nanocrystals HPAEC-PAD chromatogram of C1 oxidizing LPMO incubated with cellulose nanocrystals for 45 minutes in the presence of 500 μM L-ascorbic acid. The identity of D-glucose (G1), D-cellobiose (G2), L-ascorbic acid (Asc) and D-gluconic acid (A1) were assigned using authentic standards.

Supplementary References

1. Payne, C. M. *et al.* Fungal cellulases. *Chem. Rev.* **115,** 1308–1448 (2015).
2. Igarashi, K. *et al.* Traffic jams eeduce hydrolytic efficiency of cellulase on cellulose surface. *Science* **333,** 1279–1282 (2011).
